# Association between sitting time and non-alcoholic fatty live disease in South Korean population: a cross-sectional study

**DOI:** 10.1186/s12944-020-01385-6

**Published:** 2020-09-23

**Authors:** Jae Hong Joo, Hwi Jun Kim, Eun-Cheol Park, Sung-In Jang

**Affiliations:** 1 Department of Public Health, Graduate School, Yonsei University, Seoul, Republic of Korea; 2 Institute of Health Services Research, Yonsei University, 50 Yonsei-ro, Seodaemun-gu, Seoul, 03722 Republic of Korea; 3 27th Infantry Division Medical Dispensary Operation Branch, Hwacheon, Republic of Korea; 4 Department of Preventive Medicine, Yonsei University College of Medicine, 50 Yonsei-ro, Seodaemun-gu, Seoul, 03722 Republic of Korea

**Keywords:** Non-alcoholic fatty liver disease, Hepatic steatosis index, Sedentary, Sitting time, Physical activity, Obesity, South Korea

## Abstract

**Background:**

To examine the association between sitting time and non-alcoholic fatty liver disease among South Koreans aged ≥20 years.

**Methods:**

Data from the 2016–2018 Korea National Health and Nutrition Examination Survey were used for the analysis. Non-alcoholic fatty liver disease was diagnosed according to a hepatic steatosis index of > 36. Sitting time was categorized into as Q1, Q2, Q3, and Q4 using the age-adjusted quartile with Q4 being the longest sitting hour. Multiple logistic regression analysis was used to examine the association between sitting time and non-alcoholic fatty liver disease in South Korean population.

**Results:**

A total of 13,518 participants were enrolled. The odds for having NAFLD in Q1, Q2, Q3, and Q4 (sitting hours) were 1.07 (CI: 0.88–1.31), 1.16 (CI: 1.96–1.41), and 1.34 (CI: 1.11–1.61), respectively. The odds ratio increased in magnitude with longer hours of sitting time (test for trend: *P*-value = 0.0002).

**Conclusion:**

Advising physical exercises and discouraging sedentary activities may help to alleviate NAFLD among the South Korean population.

## Background

Non-alcoholic fatty liver disease (NAFLD) is among the most common liver disorders, with approximately 9–30% of the population in developed countries having NAFLD [1, 2]. Fatty liver is often associated with heavy alcohol intake, but it may also occur in the absence of alcohol. The impact of globalization has led to a more westernized lifestyle and an increased frequency of obesity, and this contributed to an increased prevalence of NAFLD in the Asian countries [3, 4]. The incidence of NAFLD is projected to increase due to the prevalence of sedentary behavior. If left untreated, NAFLD leads to end-stage liver disease. Thus, NAFLD is a serious public health burden that needs to be addressed and managed immediately [5].

Based on an evolutionary perspective, moving and engaging in every manner of manual labor was essential to survival of our species [6, 7]. However, technological advances have created environments where sedentary lifestyle is encouraged and this is an increasing public health concern [8]. The prevalence of sedentary behavior is increasing in modern societies, and adults are becoming less active throughout the day [9]. Sedentary behavior involves sitting or lying down and not sufficiently spending energy substantially during the awake time [10]. There is increasing evidence that excessive sitting time is associated with higher risk for adverse health outcomes such as cardiovascular disease, type 2 diabetes, cancer, and mortality [11–13]. Maintaining balanced energy expenditure and physical activity is a key aspect of healthy behavior; thus, sedentary lifestyle should not be considered lightly.

A positive relationship between sedentary behavior and NAFLD has been reported persistently. NAFLD is closely linked to obesity, insulin resistance, metabolic disorders, and all of which are associated with excessive sedentary behavior [14, 15]. Previously, a cross-sectional study conducted among the Chinese workers suggested that the positive association between sitting time and the prevalence NAFLD might be affected by inflammation [16]. The possible explanation for this finding might be the reduced circulating levels of aminotransferases and decreased hepatic steatosis mechanism linked to sedentary status [17]. Furthermore, other studies also demonstrated a close relationship between sedentary lifestyle and NAFLD [18–20]. These studies suggested that prolonged sitting time and decreased physical activity level were positively associated with the prevalence of NAFLD, supporting the importance of reducing time spent sitting in addition to promoting physical activity.

The purpose of this study is to examine the association between sitting time and NAFLD in the South Korean population and to elucidate whether the prolonged sitting time plays a potential role in developing NAFLD.

## Methods

### Study participants

Data were obtained from the 2016–2018 Korea National Health and Nutrition Examination Survey (KNHANES), which was conducted by the Korea Centers for Disease Control and Prevention. The KNHANES is a self-report survey conducted in Koreans of all age and is designed to gather annual national data on sociodemographic, economic, and health-related conditions and behaviors. The survey is consisted of three components (health interview, health examination and nutrition survey), all of which are conducted by trained staff members including physicians and medical technicians [21].

The health interview and health examination are performed by trained medical staff and interviewers at the mobile examination center. One week after the health interview and health examination surveys, dieticians visit the homes of participants for the nutrition survey. The food frequency questionnaire is composed of 63 food items that are key sources of energy and nutrients. The food intake questionnaire has been designed as an open-ended survey for reporting various dishes and foods using the 24-h recall method with various measuring aids.

**Fig. 1:**
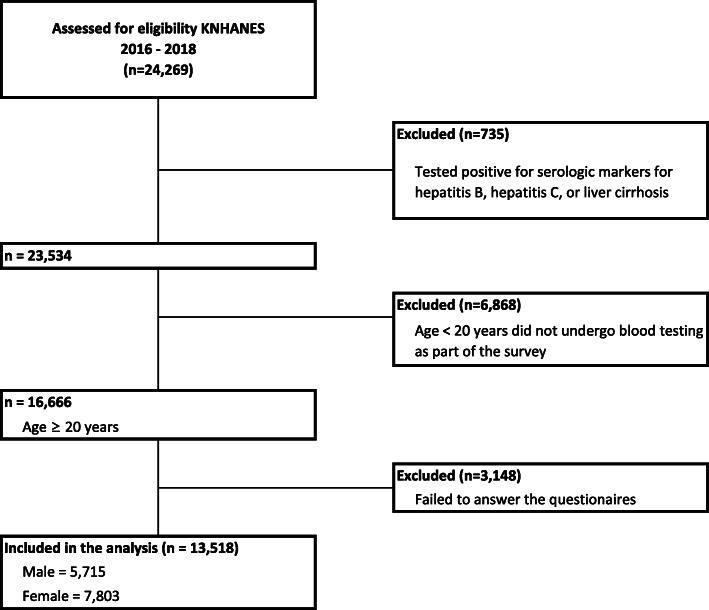
Flow diagram of subject inclusion and exclusion

Of the 24,269 survey participants, the study excluded who tested positive for serologic markers for liver disease (hepatitis B, hepatitis C, or liver cirrhosis) (*n* = 735), were aged < 20 years who did not undergo blood testing conducted by the KNHANES (*n* = 6868), and were not representative of covariates considered in the study (failed to answer the survey questionnaires) (*n* = 3148). Accordingly, the final sample size consisted of 13,518 participants (Fig. 1). This study was an analysis of existing data; thus it did not require approval by ethics review board. The data that was used in this study is the KNHANES and it has been getting an annual review and approval by Korea Centers for Disease Control (KCDC) Research Ethics Review Committee since 2007.

This study investigated the effect of prolonged sitting particularly on NAFLD. The participants with FLD related to alcohol were eliminated during the process of comprising the study sample. FLD related to alcohol was determined by biochemical and clinical profiles of the participants that were examined by the KNHANES’s trained staff members (i.e. physicians and medical technicians) [21].

### NAFLD classification

NAFLD is the main dependent variable in this study. NAFLD in this study was diagnosed according to the hepatic steatosis index (HSI), which was developed by the Department of Internal Medicine and Liver Research Institute in Seoul National University College of Medicine to efficiently select individuals for liver ultrasonography ^22^. The HSI formula was derived via logistic regression model using serum alanine aminotransferase (ALT) to serum aspartate aminotransferase (AST) ratio, body mass index (BMI), and diabetes mellitus status: HSI = 8 × (ALT/AST ratio) + BMI (+ 2, if female; + 2, if with diabetes mellitus) [22]. Participants were considered to have NAFLD if their HSI value was above 36.

### Sitting time

The main independent variable is the participants’ sitting time. Sitting time was measured by asking participants to report the following question adopted from the long-version of the International Physical Activity Questionnaire (IPAQ) [23, 24]. The overall daily sitting time was estimated particularly following questions: How many hours do you typically spend sitting or lying down while engaged in activities such as working at a desk or computer, visiting friends, driving, reading, writing, watching television, playing games, using the Internet, or listening to music on a usual day? Participants’ responses to sitting time were divided into 4 categories using age-adjusted quartile (Table 1).

**Table 1:** Age-adjusted sitting time categorized using quartile

Age group	N	Sitting time (hr)					
		Q1	Median	Q3	Max	Mean	SD
20–29	1609	7	10	12	20	9.56	3.45
30–39	2167	5	8	10	18	8.32	3.47
40–49	2558	5	8	10	19	7.92	3.40
50–59	2551	5	7	10	20	7.28	3.34
60–69	2416	5	7	10	22	7.32	3.38
≥ 70	2217	6	8	12	20	8.74	3.70

### Covariates

Sociodemographic, economic, and health-related factors were also considered in the study. Sociodemographic factors included age, educational attainment, and marital status. Economic factors included household income and occupation. Health-related factors include sleeping time (hours), total energy intake ((carbohydrate(g) × 4 kcal/g) + (protein(g) × 4 kcal/g) + (fat(g) × 9 kcal/g)), daily physical activity level based on the KNHANES questionnaire which was adopted from the World Health Organization guideline (active: ≥150 min of moderate activity, ≥ 75 min of vigorous activity, or a mixture of both for ≥150 min; inactive: < 150 min of moderate activity, < 75 min of vigorous activity, or a mixture of both for < 150 min) [25], pack years of smoking, current drinking status, comorbidity of hypertension, and comorbidity of diabetes mellitus.

### Statistical analysis

**Fig. 2:**
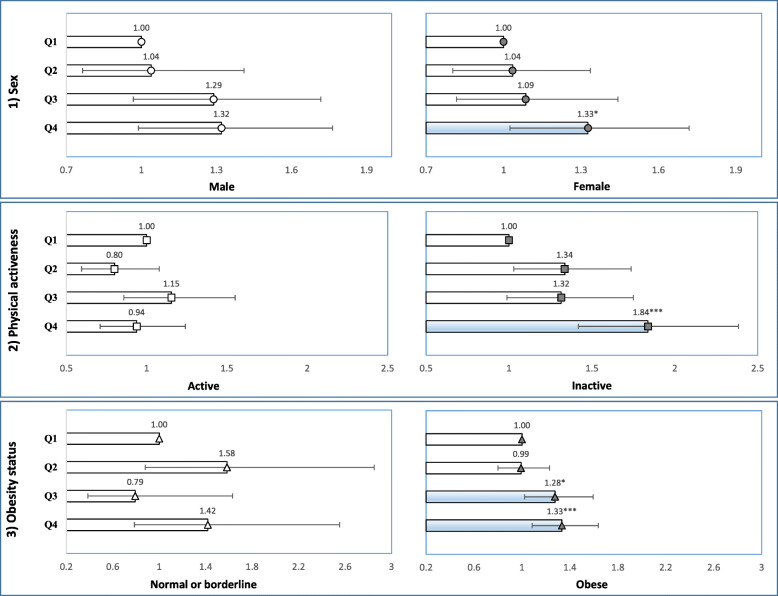
Forest plot representing odds ratio for NAFLD according to the participants’ age-adjusted sitting time stratified by 1) sex, 2) physical activeness, and 3) obesity status defined by BMI. *P-value < 0.05, ***P-value < 0.01

The frequencies and percentages of participants were calculated for each of the categorized variables included in the study. The variables included in the analysis were all categorical, those that were not initially categorical were converted into categories (age, BMI, total energy intake). The chi-square (χ^2^) test was performed to assess the chi-square differences between the groups within each categorized variable (Table 2). Multiple logistic regression analysis was used to calculate the odds ratios (with 95% confidence intervals) for NAFLD according to the participants’ report on sitting time (Table 3). The sub-group analysis for NAFLD stratified by the participants’ sex, physical activeness, and obesity status defined by BMI was also performed using multiple logistic regression (Fig. 2).

**Table 2:** General characteristics of the study population

Variables	Non-alcoholic fatty liver disease (NAFLD)						
	TOTAL		Yes^**a**^		No^**b**^		P-value*
	N	%	N	%	N	%	
**Total**	13,518	100.0	3140	23.2	10,378	76.8	
**Age-adjusted sitting time (hr)**^**c**^							<.0001
Q1 (lower < 25%)	2802	26.1	566	20.2	2236	79.8	
Q2	2816	26.3	629	22.3	2187	77.7	
Q3	3519	32.8	798	22.7	2721	77.3	
Q4 (upper > 75%)	4381	40.9	1147	26.2	3234	73.8	
**Sex**							<.0001
Male	5715	53.3	1539	26.9	4176	73.1	
Female	7803	72.8	1601	20.5	6202	79.5	
**Age**							<.0001
20–29	1609	15.0	308	19.1	1301	80.9	
30–39	2167	20.2	545	25.1	1622	74.9	
40–49	2558	23.9	615	24.0	1943	76.0	
50–59	2551	23.8	633	24.8	1918	75.2	
60–69	2416	22.5	597	24.7	1819	75.3	
≥ 70	2217	20.7	442	19.9	1775	80.1	
**Marital status**							0.0443
Married and living together	9499	88.6	2177	22.9	7322	77.1	
Living apart, divorced, or deceased	1781	16.6	455	25.5	1326	74.5	
Unmarried	2238	20.9	508	22.7	1730	77.3	
**Educational attainment**							<.0001
≤ Highschool	8350	77.9	2048	24.5	6302	75.5	
≥ College	5168	48.2	1092	21.1	4076	78.9	
**Occupation**^**d**^							0.0099
White-color	3429	32.0	750	21.9	2679	78.1	
Blue-color	3106	29.0	764	24.6	2342	75.4	
Pink-color	1716	16.0	432	25.2	1284	74.8	
Unemployed	5267	49.2	1194	22.7	4073	77.3	
**Household income**							<.0001
Low	2472	23.1	612	24.8	1860	75.2	
Mid-low	3290	30.7	803	24.4	2487	75.6	
Mid-high	3739	34.9	896	24.0	2843	76.0	
High	4017	37.5	829	20.6	3188	79.4	
**Sleeping time (hr)**							<.0001
< 7	5010	46.8	1256	25.1	3754	74.9	
≥ 7	8508	79.4	1884	22.1	6624	77.9	
**Energy intake (kcal)**^**e**^							0.9991
Quintile 1 (lower < 20%)	2703	25.2	623	23.0	2080	77.0	
Quintile 2	2704	25.2	627	23.2	2077	76.8	
Quintile 3	2703	25.2	628	23.2	2075	76.8	
Quintile 4	2705	25.2	631	23.3	2074	76.7	
Quintile 5 (upper > 80%)	2703	25.2	631	23.3	2072	76.7	
**Physical activeness**							0.0017
Active	5913	55.2	1297	21.9	4616	78.1	
Inactive	7605	71.0	1843	24.2	5762	75.8	
**Pack years of smoking**							<.0001
≥ 5 packs	4884	45.6	1265	25.9	3619	74.1	
< 5 packs	275	2.6	65	23.6	210	76.4	
Never	8359	78.0	1810	21.7	6549	78.3	
**Current drinking status**							0.0019
2–4 times / week	2973	27.7	647	21.8	2326	78.2	
2–4 times / month	3047	28.4	665	21.8	2382	78.2	
Never or occasionally	7498	70.0	1828	24.4	5670	75.6	
**Obesity status defined by BMI**^**f**^							<.0001
Obese (≥25 kg/m^2^)	6765	63.1	2963	43.8	3802	56.2	
Normal or borderline (< 25 kg/m^2^)	6753	63.0	177	2.6	6576	97.4	
**Hypertension**							<.0001
Hypertension	4298	40.1	1454	33.8	2844	66.2	
Prehypertension	3260	30.4	867	26.6	2393	73.4	
Normal	5960	55.6	819	13.7	5141	86.3	
**Diabetes**							<.0001
Diabetes	1673	15.6	843	50.4	830	49.6	
Impaired fasting glucose	3253	30.4	982	30.2	2271	69.8	
Normal	8592	80.2	1315	15.3	7277	84.7	
**Year**							0.0293
2016	4347	40.6	1065	24.5	3282	75.5	
2017	4511	42.1	998	22.1	3513	77.9	
2018	4660	43.5	1077	23.1	3583	76.9	

Hepatic steatosis index = *8 x (ALT/AST ratio) + BMI (+ 2, if female; + 2, if diabetes mellitus))*

^a^Hepatic steatosis index ≥36

^b^Hepatic steatosis index < 36

^c^Age-adjusted sitting-time categorized using quartile (see Table 1)

^d^Categories based on International Standard Classification Occupations codes

^e^Total energy intake = (carbohydrate(g) × 4 kcal/g) + (protein(g) × 4 kcal/g) + (fat(g) × 9 kcal/g);

^f^Obesity status defined by BMI based on 2014 Clinical Practice Guidelines for Overweight and Obesity in Korea

*Measure for chi-squire differences between groups within each category (significant level: *P-value* < 0.05)

**Table 3:** Odds ratio for NAFLD

Variables	Non-alcoholic fatty liver disease (NAFLD)				
	OR^**a**^	95% CI	***β***	SE	***P***-value
**Age-adjusted sitting time (hr)**^**b**^					
Q1 (lower < 25%)	1.00		Ref.		
Q2	1.07	(0.88–1.31)	0.07	0.10	0.4968
Q3	1.16	(0.96–1.41)	0.15	0.10	0.1184
Q4 (upper > 75%)	1.34	(1.11–1.61)	0.29	0.09	0.0022
*Test for trend*	*P-value =* 0.0002				
**Sex**					
Male	1.00		Ref.		
Female	0.91	(0.76–1.09)	− 0.09	0.09	0.3063
**Marital status**					
Married and living together	1.00		Ref.		
Living apart, divorced, or deceased	1.09	(0.90–1.33)	0.09	0.10	0.3894
Unmarried	1.25	(0.97–1.60)	0.22	0.13	0.0801
**Educational attainment**					
≤ Highschool	1.08	(0.93–1.27)	0.08	0.08	0.3195
≥ College	1.00		Ref.		
**Occupation**^**c**^					
White-color	1.00		Ref.		
Blue-color	0.95	(0.79–1.15)	−0.05	0.10	0.6055
Pink-color	1.19	(0.94–1.49)	0.17	0.12	0.1420
Unemployed	1.29	(1.07–1.54)	0.25	0.09	0.0067
**Household income**					
Low	1.08	(0.87–1.35)	0.08	0.11	0.4851
Mid-low	1.06	(0.90–1.26)	0.06	0.09	0.4740
Mid-high	1.18	(1.00–1.39)	0.17	0.08	0.0461
High	1.00		Ref.		
**Sleeping time (hr)**					
< 7	1.01	(0.89–1.14)	0.01	0.06	0.9237
≥ 7	1.00		Ref.		
**Energy intake**^**d**^					
Quintile 1 (lower < 20%)	0.90	(0.75–1.09)	−0.10	0.09	0.2898
Quintile 2	0.86	(0.70–1.05)	−0.16	0.10	0.1274
Quintile 3	1.00		Ref.		
Quintile 4	0.99	(0.83–1.19)	−0.01	0.09	0.9468
Quintile 5 (upper > 80%)	1.05	(0.86–1.28)	0.05	0.10	0.6521
**Physical activeness**					
Active	1.00		Ref.		
Inactive	1.31	(1.16–1.48)	0.27	0.06	<.0001
**Pack years of smoking**					
≥ 5 packs	0.99	(0.84–1.18)	−0.01	0.09	0.9391
< 5 packs	0.93	(0.59–1.45)	− 0.08	0.23	0.7371
Never	1.00		Ref.		
**Current drinking status**					
2–4 times / week	0.50	(0.42–0.59)	−0.70	0.09	<.0001
2–4 times / month	0.75	(0.64–0.88)	− 0.29	0.08	0.0005
Never or occasionally	1.00		Ref.		
**Obesity status defined by BMI**^**e**^					
Obese (≥25 kg/m^2^)	38.93	(31.62–47.92)	3.66	0.11	<.0001
Normal or borderline (< 25 kg/m^2^)	1.00		Ref.		
**Hypertension**					
Hypertension	2.29	(1.93–2.73)	0.83	0.09	<.0001
Prehypertension	1.85	(1.58–2.17)	0.62	0.08	<.0001
Normal	1.00		Ref.		
**Diabetes**					
Diabetes	7.19	(5.91–8.73)	1.97	0.10	<.0001
Impaired fasting glucose	1.90	(1.65–2.19)	0.64	0.07	<.0001
Normal	1.00		Ref.		
**Year**					
2016	1.00		Ref.		
2017	0.37	(0.32–0.44)	−0.98	0.09	<.0001
2018	0.40	(0.35–0.47)	−0.91	0.08	<.0001

Hepatic steatosis index = *8 x (ALT/AST ratio) + BMI (+ 2, if female; + 2, if diabetes mellitus))*

^a^Hepatic steatosis index ≥36

^b^Age-adjusted sitting-time categorized using quartile

^c^Categories based on International Standard Classification Occupations codes

^d^Total energy intake = (carbohydrate(g) × 4 kcal/g) + (protein(g) × 4 kcal/g) + (fat(g) × 9 kcal/g);

^e^Obesity status defined by BMI based on 2014 Clinical Practice Guidelines for Overweight and Obesity in Korea

The reported odds ratios were adjusted for all covariates considered in the study. The sampling weight variables were applied in the analysis to improve the representativeness of the sample. KNHANES has constructed sample weights to take into account survey non-response, over-sampling, post-stratification, and sampling error. The use of sample weights in the analysis is recommended to produce an unbiased national estimate. For all data analysis, SAS version 9.4 (SAS Institute, Inc., Cary, NC, USA) was used and the significance level was set at *P*-value < 0.05.

## Results

The general characteristics of the study population are shown in Table 2. Of 13,518 participants, 3140 (23.2%) reported had NAFLD. The frequencies of having NAFLD varied across the different sitting hour categories. The participants who reported the longest hours of sitting per day (Q4) had the highest frequency of having NAFLD (26.2%), whereas those who reported the shortest hours of sitting per day (Q1) had the lowest frequency of having NAFLD (20.2%) among the study sample.

Based on the odds ratios calculated from the multiple logistic analysis, the relationship between NAFLD and sitting time was analyzed along with other covariates. The participants in the Q1 group served as the reference group in this study and the relative risk for other ‘sitting time’ groups are expressed in Table 3. The odds for NAFLD in ‘Q2’, ‘Q3’, and ‘Q4’ were 1.07 (CI: 0.88–1.31), 1.16 (CI: 1.96–1.41), and 1.34 (CI: 1.11–1.61), respectively. The odds ratio increase in magnitude with longer hours of sitting time was tested for statistical significance using the linear trend test (test for trend: *P*-value = 0.0002).

Odds ratio for NAFLD according to the participants’ sitting time was stratified by sex, physical activity level, and obesity status (Fig. 2). In this analysis, ‘Q1’ group were again served as the reference group. In both sexes, those who are in the Q4 group had relatively higher odds compared to those who reported shorter amount of sitting time (male 1.32, CI: 0.99–1.76; female 1.33, CI: 1.03–1.72). When participants were stratified by their physical activeness, the significant results were only observed in physically inactive group. Those who are in the Q4 group had the highest odds (1.84, CI: 1.42–2.38) within the physically inactive group and it was also the higher when compared to their physically active courterparts. Moreover, the risk of having NAFLD was relatively high in those who are in the Q3 group (1.28, CI: 1.02–1.59) and Q4 (1.33, CI: 1.09–1.64) among the obese participants.

## Discussion

The overall findings were that there is an association of sitting time with NAFLD and the risk of having NAFLD increases in magnitude with longer hours of sitting.

Sedentary behavior, which is characterized by a lack of muscle movement, directly affects cardiometabolic function ^6^. The human body is designed to move, and previous studies reported that inactivity results in impaired lipid steatosis and insulin resistance [26, 27]. This aligns with findings of this study which suggest that a higher rate of NAFLD was observed in sedentary participants. Prolonged sitting is correlated with higher BMI, body fat, and circulatory lipids, all of which are associated with liver lipid content [28]. Cumulative fat in the mitochondria, one of the key regulators of liver pathophysiology, progresses to non-alcoholic steatohepatitis with inflammation [29]. Mitochondrial dysfunction disrupts the encoding of cytochromes during carcinogenesis, and this leads to increased reactive oxygen species (ROS) production. Fat disposition in the liver may be because of ROS proliferation in Kupffer cells and hepatocytes [30].

To date, lifestyle modification remains as one of the most important option in NAFLD management. The situation with respect to sedentary behavior, however, is remained widely unexplored. Thus, the present study aimed to validate the association between prolonged sitting time and NAFLD and clarify what other factors might play a potential role in developing NAFLD in the general South Korean population.

In recent years, it has been reported that sedentarism is a risk factor for numerous negative health outcomes, independent of exercise, and that increasing structured exercise time does not counteract sitting time [12, 31–33]. The present study clarified these previous findings by examining the odds for NAFLD according to the participants’ sitting time and simultaneously adjusting for the participants’ physical activeness along with other covariates. This was typically done so that the physical activity can no longer act as a confounder. This study supports the evidence that the risk for NAFLD increases in magnitude with longer hours of sitting. Furthermore, the subgroup analysis was performed to examine the combined interactions between prolonged sitting time and physical activity on NAFLD. It seemed that the risk for NAFLD is relatively high for the people in the Q4 group who were also physically inactive. Thus, suggesting that physical inactivity in conjunction with prolonged sitting can potentially increase the probability of having NAFLD.

In this study, the probability of having NAFLD was relatively high in participants who were sedentary and physically inactive. Physical activity is the primary strategy for managing NAFLD. Gains in physical activity improves serum liver enzymes, reduces hepatic fatty infiltration, and reduces a degree of hepatic inflammation and fibrosis, all of which contribute to health benefits beyond the prevention of NAFLD [34]. The benefits of exercise and fitness have been supported by results of previous clinical trials. Normalized level of ALT and significant weight loss were observed in participants who increased their physical activity [35, 36]. Regular physical activity is important because the frequency and intensity of physical activity are strong factors for predicting a person’s hepatic outcome.

Primarily, NAFLD is a consequence of insulin resistance, and thus occurs frequently among obese participants [37]. This study’s results showed that the risk of developing NAFLD is higher in obese participants who were more likely to be sedentary. Obesity is associated with increased visceral fat that releases free fatty acids into the hepatic portal circulation and the concentration of free fatty acids serves as the key mediator of insulin resistance [38]. The absence of physical activity aggravates insulin sensitivity and glucose homeostasis. Sedentary behavior causes downregulation of insulin receptor in muscle tissue [39, 40]. This means that prolonged sitting and physical inactivity impairs the delivery of insulin and glucose to the muscle and hence deteriorate the metabolism of free fatty acid [41].

### Study strength and limitations

The strength of the study is that the dataset generated from the KNHANES is nationally representative of the health status in South Korea. KNHANES questionnaires are updated annually to incorporate the changes in the real-life health circumstances of South Koreans. KNHANES has been extremely useful in health-related studies and provides meaningful insights for South Korean health policies. This study has several limitations. First, although HSI has been proven to have high sensitivity for detecting NAFLD (sensitivity of 93.1% and specificity of 92.4%) [22], there are still tiny odds of false-positive or false negative. Second, this is study was a cross-sectional study and was unable to provide a causal relation between sitting time and NAFLD. Third, the KNHANES uses self-report questionnaires, and thus the data extracted may have been subject to recall bias.

## Conclusion

This study found that prolonged sitting time (sitting time of > 10 h/day) was associated with NAFLD among the South Korean population aged > 20 years. The adverse effect of sitting time on NAFLD was mediated by physical inactiveness and obesity. The findings of this study suggest that lifestyle modifications to engage in physical activity and reduce weight could contribute to managing and preventing NAFLD in the South Korean population. Interventions on sitting time may provide a new solution for the prevention of NAFLD in South Korea.

## Data Availability

The KNHANES was openly available in https://knhanes.cdc.go.kr/knhanes/index.do by submitting written oath and data utilization plan.
